# Building a national direction for research in the prevention of mother to child transmission of HIV: results from a national prioritization initiative in Malawi

**DOI:** 10.1186/1478-4505-11-40

**Published:** 2013-10-26

**Authors:** Megan Landes, Monique van Lettow, Fabian Cataldo, Adrienne K Chan, Beth Tippett Barr, Anthony D Harries, Richard Bedell

**Affiliations:** 1 Department of Family and Community Medicine, University of Toronto, Toronto, Canada; 2 Dignitas International, PO Box 1071 Zomba, Malawi; 3 Dalla Lana School of Public Health, University of Toronto, Toronto, Canada; 4 Faculty of Medical Sciences, University College, London, UK; 5 Division of Infectious Diseases, St. Michael’s Hospital, Toronto, Canada; 6 Centers for Disease Control and Prevention (CDC), Lilongwe, Malawi; 7 International Union Against Tuberculosis and Lung Disease, Paris, France; 8 London School of Hygiene & Tropical Medicine, London, UK

**Keywords:** Malawi, National agenda, PMTCT Option B+, Research priorities

## Abstract

**Background:**

In 2011, Malawi initiated an ambitious program for the prevention of maternal to child transmission (PMTCT) of HIV, called 'Option B+,’ which employs a universal test and life-long treatment strategy for all pregnant women. Priority setting should take place in defining a national research agenda for evaluating Option B + rollout in Malawi.

**Methods:**

In April 2011, a three-day workshop took place for all major stakeholders in PMTCT aiming to provide an update on current PMTCT operational research in Malawi, find consensus on key questions not yet being addressed, identify opportunities for collaboration, and develop multi-partner research proposals.

**Results:**

Overall, 24 participants attended the workshop including representatives from the Ministry of Health, the National AIDS Commission and 12 multilateral, non-governmental organizations and academic partners.

Three interrelated clusters emerged as priorities for research: i) pregnancy intentions and family planning needs; ii) evaluation of models of care; and iii) determinants of uptake, adherence, and retention of women for Option B+. In addition, two cross-cutting themes arose: partner involvement in PMTCT services and cost-effectiveness as a guide to priority setting.

Within each cluster a coordinator was designated and a proposed plan for research and potential collaborators were discussed. The results of the workshop were presented to the national technical working groups and the National AIDS Commission. Several large-scale, collaborative proposals have been developed and funded to address the research areas defined.

**Conclusions:**

Option B + represents a significant change in PMTCT policy in Malawi and the process for evaluation of the Malawi PMTCT strategy is outlined. This workshop contributed to defining and coordinating the national agenda for research priorities.

## Background

Preventing mother to child transmission (PMTCT) of HIV is one of the most difficult challenges of the response to the global HIV epidemic. In 2011, the Joint United Nations Programme on HIV/AIDS (UNAIDS) estimated that 330,000 new HIV infections occurred through vertical transmission [1]. Morbidity and mortality amongst HIV-infected infants and children is high: an estimated 50% die by their second year [2].

Like other countries in sub-Saharan Africa hit hard by the HIV epidemic, Malawi (population 15 million, adult sero-prevalence 10.8% [as per latest figures UNAIDS 2013, Dr A Jahn, personal communication]) has made remarkable progress in rolling out HIV services and scaling up antiretroviral therapy (ART). However, in 2010, in light of evidence questioning the effectiveness of the current PMTCT cascade of services (from HIV testing, treatment, delivery, and breastfeed recommendations to early infant diagnosis), along with the changing WHO Guidelines for PMTCT, the Malawi Ministry of Health (MoH) decided to embark on a universal test and treat strategy for all pregnant women, called “Option B+” [3]. The overall aim of the strategy is to improve uptake and retention of HIV-infected women and infants within PMTCT by streamlining treatment, as all pregnant and lactating women are offered lifelong ART regardless of CD4 count or WHO staging.

Option B + began in July 2011 and rapidly showed an increase in access to ART for pregnant and breastfeeding women as compared to the previous approach in Malawi [4]. However, as a strategy for national implementation, this was an innovative, unproven approach [5]. Alongside the MoH and the National AIDS Commission, multiple implementing partners and academic institutions in Malawi recognize the urgent need for priority setting around the evaluation of this strategy for PMTCT within Malawi.

Dignitas International, a Canadian NGO working in the south-eastern region of Malawi since 2004 as an implementing and operational research partner with the MoH, identified an opportunity to bring together national stakeholders to define research priorities for this new and novel PMTCT strategy. A workshop was held in April 2011 with the following aims: i) to update participants on current PMTCT-related operational research, ii) to find consensus on key research questions not yet being addressed, iii) to identify specific opportunities for complementary coordination of efforts and multi-site collaboration, and iv) to develop specific proposal outlines for collaborative work. We herein report on the methods and outcomes of this national workshop to define collaborative research priorities for PMTCT in Malawi.

## Methods

In April 2011, a three day workshop was organized by Dignitas International in Zomba, a town in the south-eastern region of Malawi. Invitations were issued to key stakeholders working in PMTCT including the MoH, the National AIDS Commission, multilateral partners, and over twelve NGOs and academic partners.

The first half of the workshop focused on a review of the existing literature and ongoing operational PMTCT research being done in Malawi. In advance of the workshop, participants were sent the selection of recent literature on various issues in the PMTCT cascade that was to be reviewed so that they may be appropriately prepared for this exercise. After the review, the group was directed to brainstorm, categorize and subsequently define thematic or “cluster” areas of priority for operations research. During the second half of the workshop, the participants self-selected into small groups, each covering one cluster area. The small groups were tasked with further exploring and prioritizing research questions within their cluster, using a combination of information covered in the literature review and the Malawi context expertise held by the group members. Once further research questions were fleshed out and prioritized, the small group then outlined potential proposals. The research proposal outlines were then presented for plenary discussion to the whole group. The last section of the workshop focused on a discussion of potential national collaborations for pursuing defined research clusters and a national prioritization of research questions in relation to Option B+. Next steps were discussed and lead roles assigned within each cluster. Overall, the process generated a qualitative discussion (rather than other methods of prioritizing based on 'points’ and 'voting’ used by WHO) in which consensus between partners was reached.

Finally, the results of the workshop underwent a multi-level dissemination process, including presentation to the PMTCT working group at the MoH and various higher level meetings in Lilongwe involving stakeholders and the leadership of the MoH.

## Results

There were 24 stakeholders who attended the three day workshop in Zomba. Among the attendees were the MoH, the National AIDS Commission, six multilateral and NGO implementing partners involved in program support and evaluation (i.e., Médecins Sans Frontières-Belgium, Management Sciences for Health, Dignitas International, Elizabeth Glaser Pediatric AIDS Foundation, U.S. Centers for Disease Control and Prevention, UNAIDS), and five primarily academic and research based institutions (University of North Carolina-Chapel Hill Malawi Project, Medical Research Council Clinical Trials Unit-UK, College of Medicine, DREAM Clinic, Malawi Liverpool Wellcome Trust/College of Medicine, International Union Against TB and Lung Disease).

**Figure 1:**
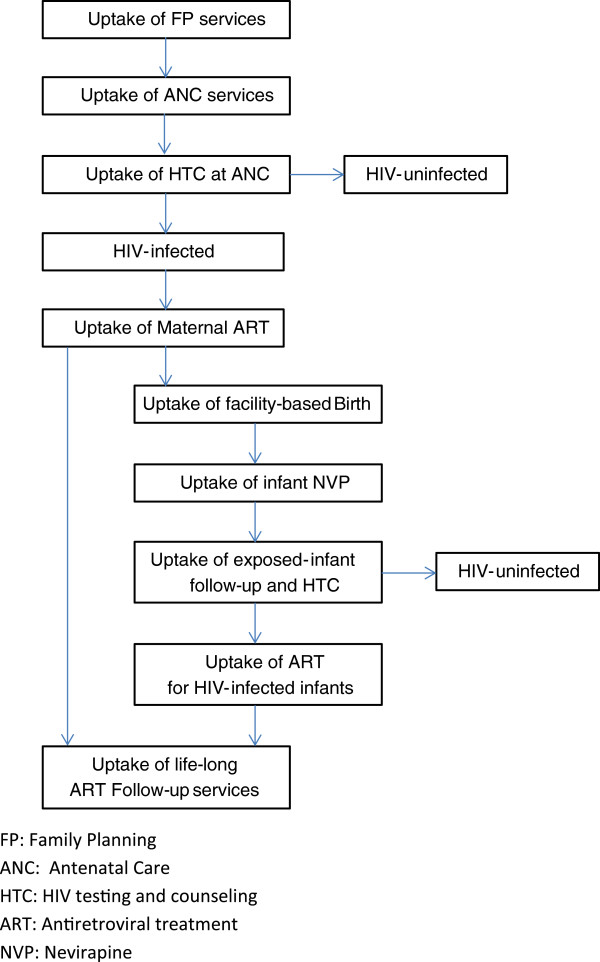
Option B+, prevention of mother-to-child transmission cascade.

Discussion considered operational research for the entire PMTCT cascade (Figure 1), from pre-pregnancy and contraception through pregnancy and lactation to follow-up of HIV-exposed and infected infants. In particular, the workshop addressed these issues in the context of Malawi’s new policy change to Option B + .

Three interrelated thematic clusters emerged as top priorities for research: i) pregnancy intentions and family planning needs; ii) models of care and evaluation of these models within the overall program structure; and iii) determinants and measurement of uptake, retention, and adherence to ART. In addition, two cross-cutting themes arose which are of relevance to all of the issues above: male involvement in HIV prevention, care, and treatment, and cost-effectiveness.

Within each cluster, the three subgroups worked to define research priorities (Table 1).

**Table 1:** Research priorities identified within clusters

**Cluster 1**	**Pregnancy intentions, family planning needs and contraceptive choices**
	▪ The feasibility, uptake and outcomes of integrated HIV and family planning services
	▪ Role of provider-initiated HIV-testing by family planning providers
	▪ Family planning preferences in relation to HIV-status of women and partners
	▪ Policy analysis of family planning implementation in the context of Option B + and the use of current models of care for the integration of family planning and PMTCT
	▪ Access to family planning services postpartum
**Cluster 2**	**Models of care and their relation to initiation of treatment, comprehensive care for women living with HIV and linkage to infant diagnosis and treatment**
	▪ A typology of possible PMTCT service delivery models and a framework for classifying each service delivery
	▪ National level aggregate outcomes related to access, uptake, adherence, retention, and degree of technical assistance at sites
	▪ Individual level data to describe outcomes related to HIV-free survival, access, uptake, adherence and retention in relation to service delivery models
	▪ Enhanced monitoring of maternal and child outcomes through development of a set of specific indicators
**Cluster 3**	**Uptake, retention in care, and adherence to treatment**
	▪ Nationally aggregated and individual level data on uptake, adherence, and retention levels during pregnancy, breastfeeding, and post-breastfeeding
	▪ Determinants of poor (and good) uptake, adherence, and retention across the PMTCT cascade
	▪ Evaluation of innovative models for support for mothers in Option B + for uptake, adherence, and treatment through antenatal, delivery, and postpartum periods

Within Cluster One, i.e., '*pregnancy intentions and family planning needs’*, participants felt that family planning was an important, yet overlooked part of routine care for people living with HIV and their partners. Family planning services were identified as an additional access point for HIV testing and counselling, in particular for those women having previously tested HIV-negative. Participants identified a lack of information regarding the current patterns of family planning service delivery in Malawi and they highlighted the rollout of Option B + as an opportunity to proactively address family planning issues. The group consensus was that this cluster’s priorities were well-defined and feasible to fund in their current form.

**Figure 2:**
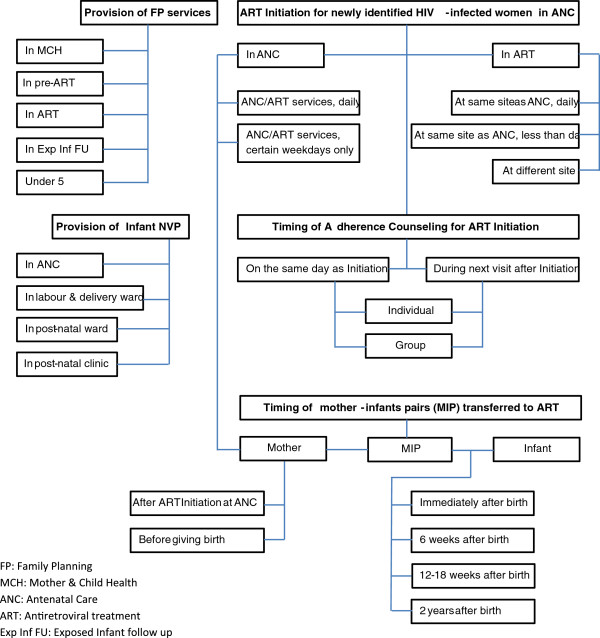
Models of care in health facilities providing PMTCT Option B + in Malawi.

Regarding Cluster Two, i.e., *'the models of care for Option B+*’, participants felt this work was most urgently related to the imminent rollout of Option B + (in July 2011) and would require close collaboration with the MoH. First, a typology of different models of care was developed in order to create a framework for assessing the development of services for PMTCT throughout Malawi as it was felt that Option B + may be more or less successful at individual site level depending on the capacity and organization of those sites (Figure 2). Second, participants highlighted the importance of effective and enhanced monitoring and evaluation at a national level. Proposals were developed for evaluation of routine programme data to describe national aggregate outcomes as well as for an enhanced collection of individual level data from a nationally representative sample in relation to Option B + and the service delivery models. Finally, the group defined a set of enhanced maternal and child indicators to be collected as individual level data in relation to uptake, retention, and clinical outcomes within the PMTCT cascade.

Within Cluster Three, i.e., '*uptake, retention in care, and adherence to treatment’,* the group felt this to be an extremely important component in the evaluation of Option B + as a long-term universal test and treat strategy for all pregnant women. The group proposed a multi-phased approach, beginning with a more thorough review of existing Malawi-specific data and followed by a mixed-methods situational analysis to define baseline adherence rates, as well as current barriers, both individual and system-wide, affecting uptake, adherence, and retention. The second proposed phase would be to implement and evaluate individual-oriented, family-oriented, and community-based support mechanisms to improve measures of uptake, adherence, and retention.

For each cluster a lead coordinator was designated and potential collaborators discussed. The results of the workshop were presented to the PMTCT technical working group on HIV, reproductive health group, and National AIDS Commission in the following six months. Participant interviews highlighted the importance of this workshop in defining a national agenda for research and building collaborative networks.

To date, several large-scale national collaborative proposals have been submitted and funded to address several of the cluster research areas. In particular, Cluster Two and Three priorities currently have collaborative projects underway. One example is a large, national collaboration, supported by the WHO and the Canadian International Development Agency to implement and evaluate innovative individual, facility-based, and community-based support models for women and their families within Option B + and their association with uptake, retention, and adherence. A second example is a national evaluation of Option B + lead by the MoH, the U.S. Centers for Disease Control, and several implementing research NGOs to measure HIV transmission, as well as follow longer term maternal and child outcomes for those women within Option B + .

Additionally, partners such as Dignitas International report having incorporated these defined priority areas into their organizational evaluation of ongoing programs. For example, Dignitas has launched an internal evaluation of the models of care development in the south-eastern region of Malawi as part of an enhanced monitoring and evaluation program.

## Discussion

Option B + is an ambitious PMTCT policy adopted by the MoH in Malawi and aimed at dramatically reducing the impact of HIV on maternal and child health outcomes. Stakeholders throughout Malawi are considering the impact of this policy at individual and population levels, as well as organizationally through program implementation and as a national strategy. In response to the rollout of Option B + in Malawi, we describe a national prioritization initiative which gathered a number of key stakeholders to address research in PMTCT.

First, we identified key strengths of this workshop, beginning with how the process enabled participants to look broadly at PMTCT through a sexual and reproductive health lens, reflecting the diversity of the participating stakeholders. To promote cohesion of discussion, a common evidence base was referred to, key literature was provided beforehand, and discussions allowed experts to bring in other relevant research findings during the workshop, including discussion of quantitative and qualitative research themes.

In addition, the workshop attempted to ensure a comprehensive approach to PMTCT and did not focus on 'Option B+’ in strict isolation. The focus was optimization of PMTCT given the new policy and support for the MoH decision, and participants focused on the broader priorities for PMTCT within this new context.

Second, the anticipated challenges with the rapidly approaching implementation of Option B + motivated partners to come together for the purpose of finding consensus on operational research priorities, including key areas of monitoring and evaluation at an individual program and national level. Immediate priorities were set in relation to the rollout, and medium and longer term knowledge needs were defined (i.e., adherence and retention in care).

Third, consensus was robust partly because of the full range of organizational stakeholders involved. This created an opportunity for dialogue on prioritization between key stakeholders who would usually 'compete’ for resources and not necessarily discuss collaboration. The meeting helped to clarify what areas of research partners were currently engaged in, what they could bring to collaboration (e.g., technical capacities) and how they could provide direction for future collaboration.

Finally, the dissemination process of the workshop allowed for further input and involvement from stakeholders. The preparation of the workshop report involved the opportunity for feedback and editing by participants, and dissemination was strategically planned to include face-to-face discussions between certain experts present and MoH representatives, in addition to dissemination of the printed report. The report was presented in full but with a concise version for quick review and a PowerPoint slide set to facilitate presentations.

This workshop marks the first time such an agenda has been set amongst those key stakeholders in PMTCT in Malawi. The outcomes of the workshop have contributed to defining the PMTCT research agenda around Option B+, and have provided both an impetus for and direction to national collaborative projects between key national stakeholders. This fundamental groundwork allowed for a quick response to granting opportunities and successful outcomes within that process through the strength of the collaboration.

## Conclusions

Option B + represents a dramatic change in PMTCT policy for Malawi and we describe an innovative exercise engaging key stakeholders in a national priority setting for research within this evolving context. Key ideas discussed during the workshop were further developed and funded. Collaboration between stakeholders has been enhanced as demonstrated by the partnerships on new grant applications and subsequent meetings and consortia have been formed to develop these projects.

Key questions remain as Option B + is now planned or underway in over a dozen more countries, and a similar prioritization exercise should be conducted at an international level in order to explore the range of emerging issues in various contexts and to promote collaborative and innovative approaches to research around this new intervention.

## Abbreviations

ART: Antiretroviral therapy; MoH: Ministry of Health; PMTCT: Preventing mother to child transmission; UNAIDS: Joint United Nations Programme on HIV/AIDS NGO: Non governmental organization.

## Competing interests

The authors declare that they have no competing interests.

## Authors’ contributions

All authors contributed to the concept and the design of the workshop. RB was the primary organizer and chair for the workshop. ML, MvL, and RB drafted the manuscript. All authors reviewed, edited, and approved the final manuscript.
